# Effects of Physician-Nurse Substitution on Clinical Parameters: A Systematic Review and Meta-Analysis

**DOI:** 10.1371/journal.pone.0089181

**Published:** 2014-02-24

**Authors:** Nahara Anani Martínez-González, Ryan Tandjung, Sima Djalali, Flore Huber-Geismann, Stefan Markun, Thomas Rosemann

**Affiliations:** Institute of Primary Care, University of Zurich, Zurich, Switzerland; Iran University of Medical Sciences, Iran (Republic of Islamic)

## Abstract

**Background:**

Physicians’ shortage in many countries and demands of high-quality and affordable care make physician-nurse substitution an appealing workforce strategy. The objective of this study is to conduct a systematic review and meta-analysis of randomised controlled trials (RCTs) assessing the impact of physician-nurse substitution in primary care on clinical parameters.

**Methods:**

We systematically searched OVID Medline and Embase, The Cochrane Library and CINAHL, up to August 2012; selected peer-reviewed RCTs comparing physician-led care with nurse-led care on changes in clinical parameters. Study selection and data extraction were performed in duplicate by independent reviewers. We assessed the individual study risk of bias; calculated the study-specific and pooled relative risks (RR) or weighted mean differences (WMD); and performed fixed-effects meta-analyses.

**Results:**

11 RCTs (N = 30,247) were included; most were from Europe, generally small with higher risk of bias. In all studies, nurses provided care for complex conditions including HIV, hypertension, heart failure, cerebrovascular diseases, diabetes, asthma, Parkinson’s disease and incontinence. Meta-analyses showed greater reductions in systolic blood pressure (SBP) in favour of nurse-led care (WMD −4.27 mmHg, 95% CI −6.31 to −2.23) but no statistically significant differences between groups in the reduction of diastolic blood pressure (DBP) (WMD −1.48 mmHg, 95%CI −3.05 to −0.09), total cholesterol (TC) (WMD -0.08 mmol/l, 95%CI -0.22 to 0.07) or glycosylated haemoglobin (WMD 0.12%HbAc1, 95%CI -0.13 to 0.37). Of other 32 clinical parameters identified, less than a fifth favoured nurse-led care while 25 showed no significant differences between groups.

**Limitations:**

disease-specific interventions from a small selection of healthcare systems, insufficient quantity and quality of studies, many different parameters.

**Conclusions:**

trained nurses appeared to be better than physicians at lowering SBP but similar at lowering DBP, TC or HbA1c. There is insufficient evidence that nurse-led care leads to better outcomes of other clinical parameters than physician-led care.

## Introduction

A WHO Report showed the global number of health care providers, namely physicians, nurses and midwives, remains lower than required per 1,000 population [1]–[3]. The low number of physicians, changes in working culture and trends in retirement have contributed greatly to this shortage [4]. Furthermore, there are pressing demands for high-quality affordable care due to the escalating growth and ageing of the population, patients’ expectations and the costs incurred managing complex conditions. In response to these changes and healthcare demands, the practice of skill mix has further developed with the aim of maintaining high-quality affordable and accessible care. It refers to a mix of posts, grades, occupations or employees, or to a combination of activities or skills needed for a job [5]. Of the skill mix strategies, substitution of physicians by nurses is a very appealing strategy due to its potential to address workforce shortages, maldistribution of workload, and to reduce cost [2], [6]. Substitution refers to nurses both performing tasks and taking responsibility for care that formerly would have been performed by physicians alone. Two systematic reviews published in 2002 and 2005 found no appreciable differences between nurse-led care and physician-led care on health outcomes but there were only a small number of studies and these also had methodological limitations [7], [8]. We performed a systematic review to compare the effectiveness of nurse-led care and physician-led care on clinical parameters in studies in which nurses substituted physicians.

## Methods

We developed a protocol prior to the commencement of the review and followed the PRISMA guidelines [9] for the reporting of systematic reviews (Checklist S1).

### Study Inclusion and Exclusion Criteria

We included peer reviewed randomised controlled trials (RCTs) published in English from any country that examined physician-nurse substitution. The studies had to focus on patients of all ages seeking first contact or undergoing care for all conditions including mental health and addiction restricted to primary care; and had to compare care from nurses to care from physicians (family physicians, paediatricians and geriatrician). We further limited the inclusion criteria to studies: in which the intervention or follow-up care had taken place in general practices, community or ambulatory care settings regardless of the recruitment sources; and which reported on clinical parameters that detected changes in the clinical status and/or physiological capability of patients in relation to various forms of disease, e.g. blood pressure for hypertension or cardiovascular disease risk. Based on a published framework [8], we excluded trials where nurses either supplemented the work of physicians (i.e. complemented or extended care) or collaborated with other clinicians and the effect of the intervention between nurse and physician could not be distinguished. We excluded measures of quality of life, satisfaction, mortality, hospital admissions, and progression of disease and process of care.

### Study Identification

We comprehensively searched OVID Medline, Embase, The Cochrane Library of Systematic Reviews, CINHAL and the Cochrane Effective Practice and Organisation of Care Group (EPOC) from all available dates until August 2012. The searches - not age-, date- or country-specific - included ‘primary care’, ‘skill mix’, ‘doctor’-‘nurse’ substitution’ (Table S1). We also hand-searched the reference lists of included studies and relevant reviews.

### Study Selection

Two authors independently screened titles and abstracts and assessed the full-texts of potentially eligible publications for inclusion, resolving differences through consensus.

### Data Extraction

Two authors independently extracted both qualitative data (characteristics of studies, population and interventions) and numeric data (dichotomous and continuous format) using standardised data collection forms designed and developed *a-priori,* and resolved differences through consensus. Data from more than one control group of interest (e.g. family physicians and paediatricians) were combined and compared as one to the intervention group.

### Assessment of Study Quality

Two authors independently assessed the risk of bias of individual trials following established criteria [10], [11] and resolved disagreements by consensus. A composite score was not calculated and we considered bias due to attrition of ≥20% to be of significant concern [12], [13].

### Statistical Analyses

We performed meta-analyses when at least three trials reported appropriate data for the same outcome using the generic inverse variance fixed-effects method in Review Manager (Version 5.1) [14]. We calculated the unadjusted relative risks (RR) or the weighted mean differences (WMD) of the absolute endpoint measurements. We report the summary statistics, their 95% confidence intervals (CI) and regard p<0.05 as statistically significant. We quantified heterogeneity using the I^2^ statistic: values of <25% represent low heterogeneity and ≥50% represent high heterogeneity [15]. There were a maximum of five trials per meta-analysis so we could not inspect publication bias using funnel plots [16]. We decided against further subgroup analyses due to the relatively small number of studies and small number of patients per outcome. For data not combined in meta-analyses, individual trial estimates were calculated and results were compared. If standard deviations (SD) of final measurements were unavailable and could not be calculated from the statistical analyses reported, the baseline SDs were carried forward assuming the intervention would not alter the variability of the outcome [17]. Medians were treated differently from means and are clearly stated. To ensure that all the scales pointed in the same direction, the mean of a set of studies was multiplied by −1 or the mean maximum possible value was subtracted from the scale [17].

## Results

### Study Identification

**Figure 1:**
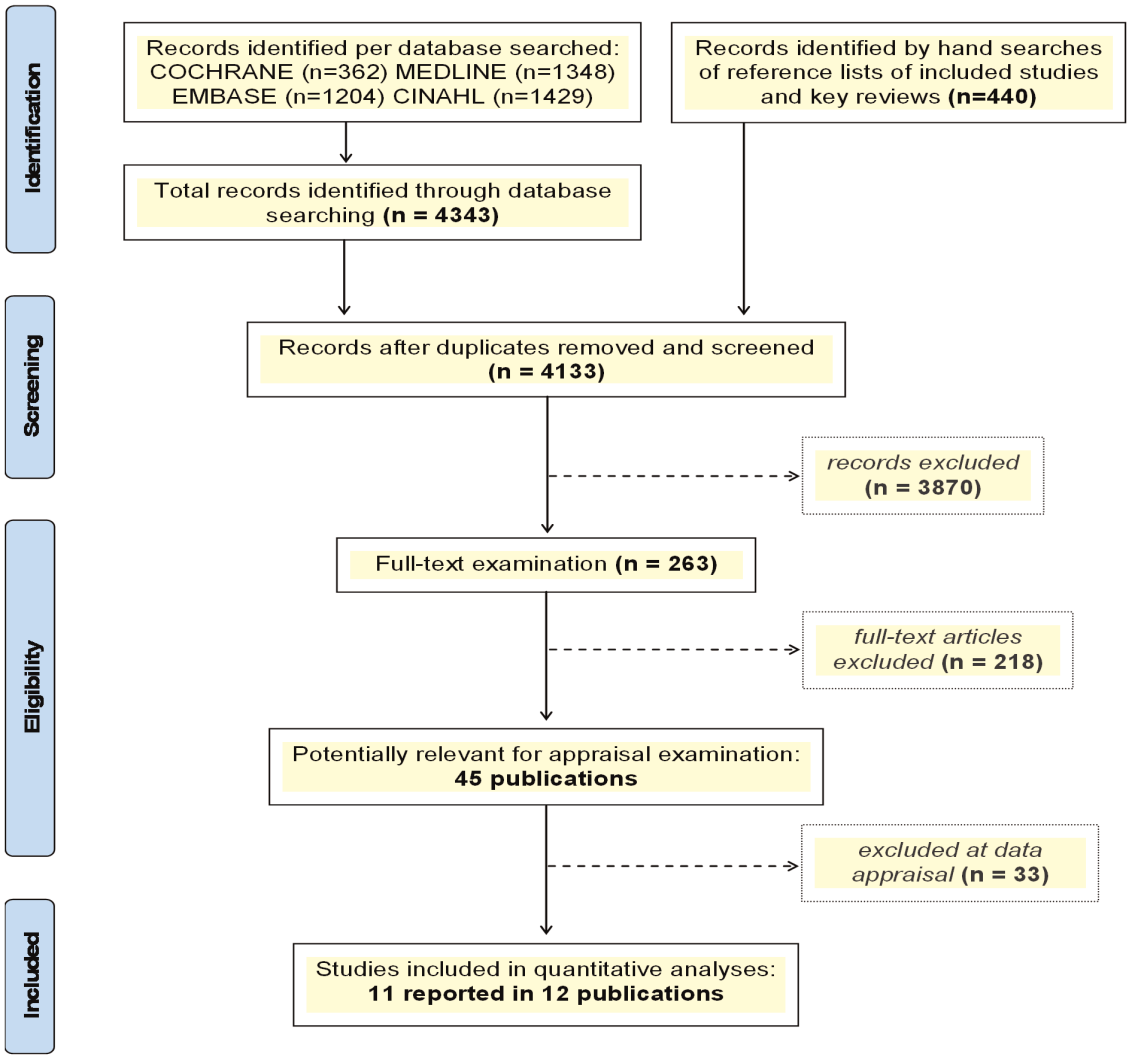
PRISMA Flow diagram – study selection process.

A total of 4,133 original records were identified (Figure 1). Forty-four publications were relevant, of which we excluded 32 for reasons provided in Table S2. In total, 11 RCTs met the inclusion criteria and comprised a total of 30,247 randomised participants, reported in twelve publications [18]–[28]. Table 1 and Table S3 show the characteristics of the populations, interventions and outcomes reported in the included studies.

**Table 1:** Characteristics of studies included in review.

Study				Setting	Participants	Nurse group				Physician’s group				Intervention delivered							Outcomes
First author, y	Location	Design, period*	FUP, m	Facilities, n	Included diagnosis	Nurses, n	Patients, N	Mean age (SD), y	Male, %	Phys., n	Patients, N	Mean age (SD), y	Male, %	By	FCA	GDL	1^st^C	UV	OC	C, n	reported
Fairall, 2012 [18].	ZA 2	cRCT2, 2008–2010.	18	Nurse ART clinic, 31.	HIV/AIDS.	103	6415	38 (8.9)	30	nr	6479	38 (9.63)	27	LN	n	y	n	n	y	>1	CD4 counts for ART continuation and regimens.
Fairall, 2012 [18].	ZA 1	cRCT1, 2008–2010.	16–18	Nurse ART clinic, 31.	HIV/AIDS.	103	6159	36 (9.6)	33	nr	4923	35 (9.63)	31	LN	n	y	n	n	y	>1	CD4 counts for ART initiation.
Houweling, 2011 [20].	NL 4	RCT, period nr.	14	Practice, 1.	DM2.	2	116	67.1 (11)	53	5	114	69.5 (10.6)	42	NP	y	y	n	n	y	>1	BP, TC, GH, TC/HDL ratio.
Kuethe, 2011 [19].	NL 3	RCT, 2006–2008.	24	Hospital outpatients, 1; Practice, 18.	Asthma.	nr	36	11.2 (2.9)	64	nr	71 (37§, 34‡)	11.2 (2.5) §; 10.1 (2.6) ‡	58	NP+	n	y	n	n	y	>1	PD20, %FEV1, FENO.
Voogdt-Pruis 2010 [21].	NL 2	RCT, 2006–2007.	12	Healthcare centre, 6.	CVD, hypertension, hypercholesterolemia.	6	808	64 (9.0)	58	25	818	64 (9.0)	62	NP+	nr	y	n	n	y	>1	BP, TC.
Andryukhin 2010 [22].	RU 1	RCT, 2006–2009.	6, 18	Medical centre practice, 1.	Heart Failure with preserved ejection fraction.	10††	50	66.5 (3.2)	27	8	50	68 (4.3)	34	NP/LN	n	y	n	n	y	>1	TC, GH, LDL, Cardiac function/inflammation.
Hiss, 2007 [23].	US 2	RCT, period nr.	6	Community, 2; PHD, 1.	DM2.	nr	95	55.7 (13.1)	32	108¶	102	57 (11.4)	35	NP+	n	y	n	n	y	>1	BP, TC, GH.
Du Moulin, 2007 [24].	NL 1	cRCT, period nr.	12	nr.	All forms of incontinence.	1 NP	38	51 (13.0)	0	28**	13	51 (13.0)	0	RN	n	y**	y	n	y	>1	Incontinence: frequency and volume.
Denver, 2003 [26].	UK 2	RCT, 2000–2001.	6	Nurse clinic hospital based.	DM2, hypertension pre-diagnosis or in receipt of BPLT.	nr	60	58.1 (13.8)	57	nr	60	62.4 (9.1)	70	NP+	n	y	n	n	y	>1	BP, TC, HG, HDL, triglycerides, kidney function.
Jarman, 2002 [27].	UK 1	RCT, 1996–1999.	24	Practice, 438†.	Parkinson’s Disease.	9	1041	nr	57	nr	818	nr	56	LN	n	nr	n	n	y	>1	Stand-up and mobility (tests).
Mundinger, 2000 [25], [28].	US 1	RCT, 1995–1997.	6–12, 24‡‡	Community clinic, 4; PC clinic, 1.	Asthma, DM, hypertension, or urgent visits.	7	1181	44	24	11	800	44.9	22	NP	n	nr	y	y	y	>1	BP, GH, peak flow.

**Legend.**

Studies are listed by year (y) of publication, in decreasing order.

Abbreviations: US = United States; NL = The Netherlands; UK = United Kingdom; ZA = South Africa; RU = Russia; RCT = Randomised Controlled Trial; cRCT = cluster Randomised Controlled Trial; FUP = follow-up; m = months; SD = standard deviation; nr = not reported; Phys.: physicians; PHD = Public health department; PC = Primary Care; ART = Antiretroviral Therapy; DM (2) = Diabetes Mellitus (Type 2); CVD = Cardiovascular Disease; BPLT = Blood Pressure Lowering Treatment; NP = nurse practitioner; NP+ = nurse practitioner with higher degree/course; RN = registered nurse; LN = licensed nurse; y = yes; n = no; FCA = full clinical autonomy; GDL = interventions based on clinical guidelines or protocols; 1^st^C. = 1^st^ contact; UV = urgent visits; OC = on-going care; n (C, n) = number of consultations; BP = blood pressure; TC = total cholesterol; GH = glycosylated haemoglobin; ART = antiretroviral therapy; CD4 = t-cell surface glycoprotein CD4; HDL = high density lipoprotein; LDL = low density lipoprotein; PD20 = provocative dose of methacholine causing a 20% fall in forced expiratory volume in one second (FEV1); FENO = fraction of exhaled nitric oxide.

^*^ start and end year when studies were conducted.

† drawn from nine randomly chosen health authority areas.

‡ paediatricians.

§ general physicians.

¶ 63 were for the control group.

^**^ 9 were physicians and 19 were supervisors; intervention delivered following clinical protocols.

†† 2 were nurse practitioners and 8 were (licensed) nurses.

‡‡ phase I follow-up: 6–12 months; phase II follow-up: 24 months.

### Study and Population Characteristics

Eleven trials - eight RCTs of parallel design and three cluster RCTs - were conducted in the UK (n = 2), The Netherlands (n = 4), USA (n = 2), South Africa (n = 2) and Russia (n = 1) (Table 1). Median follow-up was 18 (range: 6 to 30) months with more than 12 months in six trials [18]–[20], [22], [27] and 12 months or less in the other five. The number of participants ranged from 50 to 12,894 with less than 200 (range: 50 to 197) in five trials [19], [22]–[24], [26] and more than 200 (range: 230 to 12894) in the other six. Mean age reported in ten trials ranged from 11.2 (SD2.9) to 67.1 (SD11.0) years. In ten trials, 35% of the population were male and one trial included women only [24].

### Interventions

The number of nurses delivering care was reported in eight trials. It ranged from 1 to 10 in six trials. In two other trials, 31 clinics were randomised with 103 nurses. Six trials reported the number of physicians delivering care, which ranged from 5 to 28 in five trials, and another employed 108. Physicians’ resources, location of practices (e.g. rural or urban) and social settings were scarcely reported. Nurses’ years of experience were not reported in any of the trials but in most of them nurses were already enrolled as staff or took specific courses for delivering care. Nurses’ roles were described under various terminologies and their qualifications and skills varied from practice nurses with or without extra training (e.g. one week training or a specialised degree) to middle nurse managers, registered or licensed nurses.

In all studies nurses were the main figure of care and performed tasks for complex conditions that required specialised skills including cerebrovascular disease, hypertension, heart failure, diabetes mellitus, asthma, incontinence, Parkinson’s disease and HIV (Table 1). Nurses’ interventions were based on more than one consultation in all trials for patients requiring on-going care or both first contact and on-going care [24], [28] and were specifically guideline or protocol based in 82% (n = 9/11) of the trials [18]–[24], [26]. Only one trial addressed urgent visits [28]. In all trials, the physicians performed standard care. Only one trial reported that nurses had full clinical autonomy to manage patients’ disease [20]. In the other ten, there were several tasks for which nurses made independent decisions (e.g. adopting, initiating and prescribing treatment) but still needed minor support or short communication with the physicians, e.g., to discuss patients’ records, to develop action plans, and to sign prescriptions.

### Risk of Bias in the Methods of Included Studies

Table 2 summarises the risk of bias in individual studies. The quality varied substantially when assessed against current reporting standards [10], [11]. Among the included studies, 73% reported inclusion and exclusion criteria and funding sources. To measure the success of the intervention, 73% defined a primary outcome. Random sequence generation was adequate in 45.5%, allocation concealment was reported in 45.5% and both were adequate in 36.4%. No trial blinded both patients and providers. Blinded assessment of outcomes was performed in one trial only [22]. Sample size calculation was performed in 91% of the trials but only four held the least target sample required to achieve power (80% to 90%). At baseline, groups were comparable in 64% of the trials and 27% reported to have adjusted for clustering effects. Nearly half (45.5%) of the trials had an attrition rate of ≥20% (range: 11% to 54%) and only 27% used the intention to treat (ITT) techniques principle to deal with missing data.

**Table 2:** Assessment of risk of bias in studies included in review.

Study		Inclusion &exclusion criteria	Outcome		Sequencegeneration	Allocationconcealment	Blinding	Samplesize	Attrition, %	Funding
First author, y	Location		1ry	2ry						
Fairall, 2012 [18](Cohort 2)§	ZA 2	✓	✓	✓	A	A	NP‡‡	✓‡ ^,^ §§	≥20†	G
Fairall, 2012 [18](Cohort 1)§	ZA 1	✓||	✓	✓	A	A	NP‡‡	✓‡ ^,^ §§	≥20†	G
Houweling, 2011 [20]	NL 4	✓||	✓	✓	I	A	NP	✓	<20	G
Kuethe, 2011 [19]	NL 3	✓||	✓		A	A	NP	✓¶	<20	NR
Voogdt-Pruis, 2010[21]	NL 2	✓	✓		A	U	I††	✓	<20	P/Ind.
Andryukhin, 2010[22]	RU 1	✓			U	I	**	✓¶	≥20	None
Hiss, 2007 [23]	US 2	*			U	U	NP	NR	<20	G
Du Moulin, 2007 [24] §	NL 1	✓||	✓	✓	U	U	NP	✓‡ ^,^ ***	≥20	NR
Denver, 2003 [26]	UK 2	*	✓	✓	I	I	NP	✓¶	<20†	NR
Jarman, 2002 [27]	UK 1	✓	✓	✓	A	A	NP	✓	<20	P/Ind.
Mundinger, 2000[25], [28]	US 1	*			U	U	NP	✓¶	≥20	G

**Legend.**

Studies are listed by year (y) of publication, in decreasing order. A tick indicates that specific criteria were fulfilled. *Blinding*: whether patients, care providers and outcome assessors were blinded. *Attrition:* loss of data (≥20% = significant). *Intention-to-treat (ITT):* whether trial authors performed analyses (e.g. last value carried forward) to take into account all patients who began the intervention regardless of protocol violations, drop-outs or loss of follow-up [11], [12].

Abbreviations: US = United States; NL = The Netherlands; UK = United Kingdom; ZA = South Africa; RU = Russia; I = Inadequate; A = Adequate; U = Unclear; NP = Not Performed; NR = Not Reported; Funding = Government (G), Industry (Ind.) or Private (P) grant.

^*^ report the inclusion criteria only.

† used intention-to-treat (ITT) strategies but type not reported.

‡ adjusted for cluster effect or intra-class cluster correlation.

§ cluster RCT.

|| trials for which not all factors tested at baseline were comparable (i.e. ≤10% difference between groups in the factors tested).

¶ reached the least target sample required to achieve power in at least one outcome.

^**^ single blinded, nurses and physicians were not aware of patient allocation.

†† only patients were blinded.

‡‡ data analysts were partly blinded.

§§ Huber-White cluster effect approach.

^***^ intra-class cluster correlation approach not reported.

### Effectiveness of Interventions on Clinical Parameters

**Figure 2:**
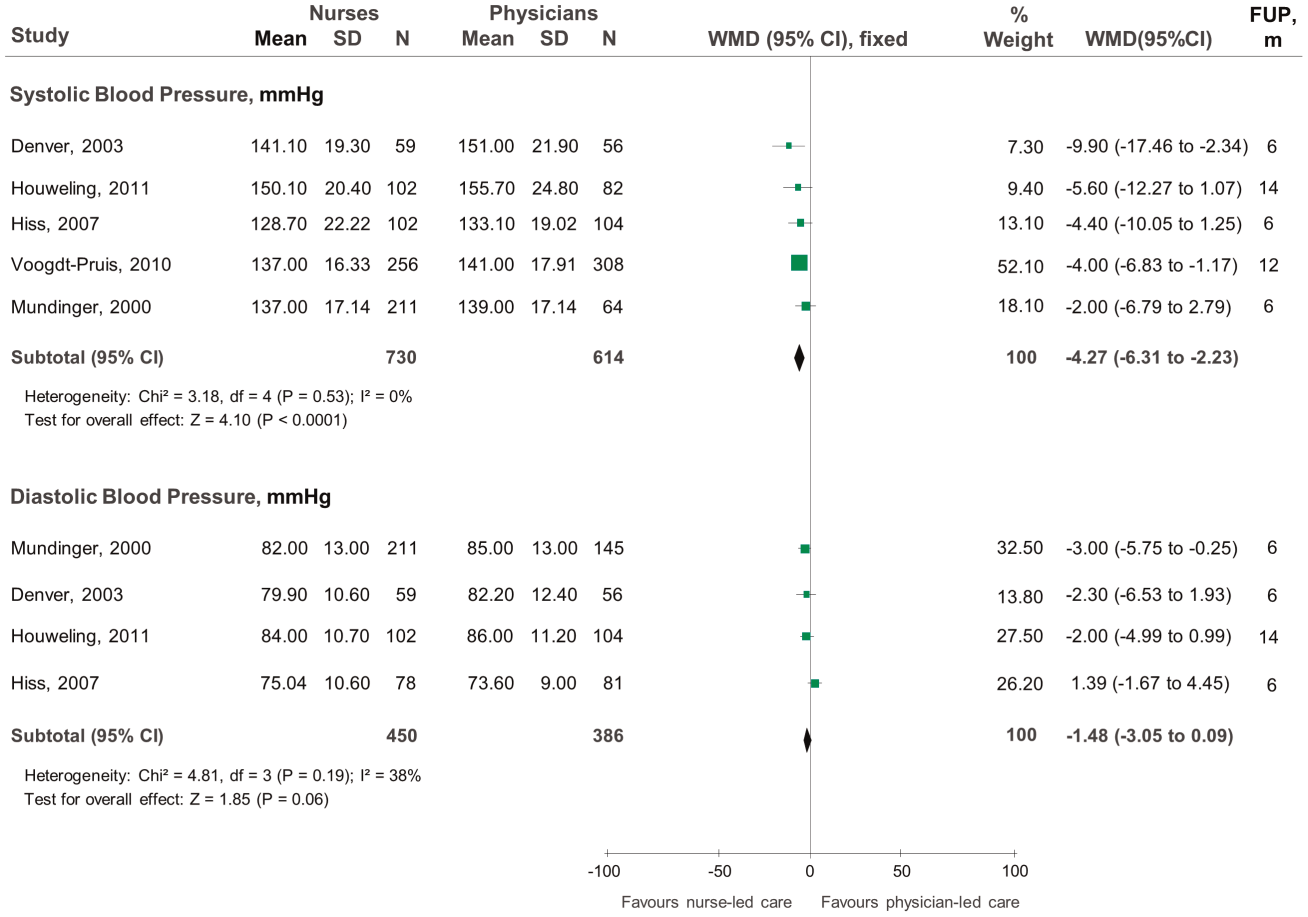
Comparison of blood pressure control between nurse-led care and physician-led care. Studies are listed in order of decreasing weighted effect size. Abbreviations: mmHg = millimetres of mercury; SD = standard deviation; N = total number of patients in the analysis; WMD = weighted mean differences; CI = confidence interval; df = degrees of freedom; I^2^ = heterogeneity between trials; FUP = Follow-up; m = months.

**Figure 3:**
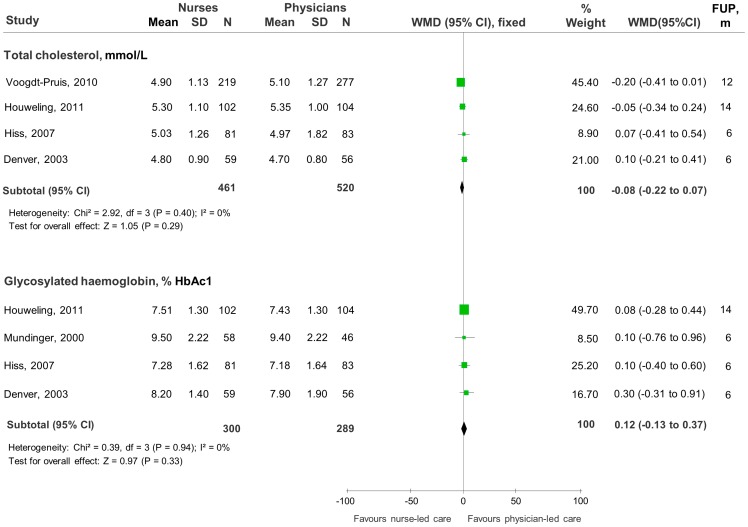
Comparison of total cholesterol and glycosylated haemoglobin control between nurse-led care and physician-led care. Studies are listed in order of decreasing weighted effect size. Abbreviations: mmol/L = millimoles per litre of blood; % HbAc1 = percent of glycosylated haemoglobin (of total haemoglobin); SD = standard deviation; N = total number of patients in the analysis; WMD = weighted mean differences; CI = confidence interval; df = degrees of freedom; I^2^ = heterogeneity between trials; FUP = Follow-up; m = months.

All RCTs [18]–[28] reported quantitative data for most of the clinical endpoints investigated but meta-analyses (Figures 2 and 3) were possible for only three, including blood pressure, systolic (SBP) and/or diastolic (DBP), total cholesterol (TC) and Glycosylated haemoglobin concentration. Thirty-two other measurements were reported in nine RCTs but had mostly one study per outcome and were not combined in meta-analyses. The individual trial estimates of these data are reported in Table 3 and Table 4.

**Table 3:** Individual trial estimates from binary data not combined in meta-analyses.

Study				Outcome		Nurse group		Physician group		Effect estimate	
First author, y			Location	Reported	FUP, m	n	N	n	N	RR (95% CI)	p
**Cholesterol triglycerides and glucose**											
Andryukhin 2010 [22] ¶			RU 1	TC regression/stay within 4.5 mmol/l.	6	23	40	10	35	2.01 (1.12 to 3.62)	0.02
Andryukhin 2010 [22] ¶			RU 1	LDL regression/stay within 2.5 mmol/l.	6	23	40	9	35	2.24 (1.2 to 4.17)	0.010
Andryukhin 2010 [22] ¶			RU 1	Glucose, decrease/regression/stay within 6 mmol/l.	6	24	40	22	35	0.95 (0.67 to 1.37)	0.800
**Cardiac function**											
Andryukhin 2010 [22] ¶		RU 1		6MWT exercise capacity decrease/regression.	6	27	40	7	35	3.38 (1.68 to 6.77)	0.001
Andryukhin 2010 [22] ¶		RU 1		NT-proBNP decrease or regression.	6	13	17	6	16	2.04 (1.03 to 4.05)	0.004
Andryukhin 2010 [22] ¶		RU 1		LASI decrease or regression.	6	27	40	19	35	1.24 (0.86 to 1.8)	0.250
Andryukhin 2010 [22] ¶		RU 1		LVEDVI decrease or regression.	6	28	40	16	35	1.53 (1.01 to 2.32)	0.040
Andryukhin 2010 [22] ¶		RU 1		LVMI decrease or regression.	6	17	40	8	35	1.86 (0.92 to 3.77)	0.090
Andryukhin 2010 [22] ¶		RU 1		E/A ratio decrease or regression.	6	19	39	14	34	1.18 (0.71 to 1.98)	0.520
Andryukhin 2010 [22] ¶		RU 1		C-reactive protein levels decrease or regression.	6	24	36	21	32	1.02 (0.72 to 1.43)	0.930
**Parkinson**’**s disease**											
Jarman, 2002 [27]	UK 1			Mobility stand-up test, unable to stand up or had tohold on.	24	329	696	247	558	1.07 (0.95 to 1.21)	0.940
Jarman, 2002 [27]	UK 1			Bone sustaining fractures during study.	24	92	696	62	558	1.19 (0.88 to 1.61)	0.690

**Legend.**

Studies are listed in order of increasing length of follow-up, within each category of outcomes.

Abbreviations: UK = United Kingdom; RU = Russia; FUP = follow-up; m = months; n = number of patients with events or number of events; N = total number of patients per group; RR = relative risk; CI = confidence intervals; LDL = Low Density Lipoprotein; TC = Total Cholesterol; NT-proBNP = N-terminal pro-brain natriuretic peptide; LASI = left atrium size index; LVEDVI = left ventricular end-diastolic volume index; LVMI = left ventricular mass index; E/A ratio = ratio of early (E) to late (A) mitral valve flow velocity; 6MWT = six minute walk test to measure of functional exercise capacity; mmol/l = millimoles per litre.

¶ positive decrease/regression corresponded to less than the upper limit of 95% CI.

**Table 4:** Individual trial estimates from continuous data not combined in meta-analyses.

Study			Outcome		Nurse group		Physician group		Effect estimate	
First author, y	Location		Reported	FUP, m	mean (SD)	N	mean (SD)	N	WMD (95% CI)	p
**Cholesterol triglycerides and glucose**										
Denver, 2003 [26]		UK 2	Mean HDL cholesterol, mmol/l.	6	1.3 (0.3)	59	1.4 (0.5)	56	−0.1 (−0.25 to 0.05)	0.200
Denver, 2003 [26]		UK 2	Mean Triglycerides, mmol/l.	6	2.4 (1.7)	59	2.3 (1.4)	56	0.1 (−0.47 to 0.67)	0.730
Voogdt-Pruis 2010 [21]		NL 2	Mean LDL cholesterol, mmol/l.	12	2.9 (1.13)	218	3.1 (1.26)	270	−0.2 (−0.41 to 0.01)	0.06
Houweling, 2011 [20]		NL 4	TC/HDL ratio.	14	4.43 (1.1)	102	4.17 (1.2)	104	0.26 (−0.05 to 0.57)	0.10
**Lung function**										
Mundinger, 2000 [28]		US 1	Mean peak flow, l/min.	6	−297 (108.05)	107	−292 (108.05)	64	−5 (−38.46 to 28.46)	0.77
Kuethe, 2011 [19]		NL 3	Mean PD20 fall in FEV1.	12	−0.12(−0.79 to 1.2)*		−0.04(−0.90 to +0.82)†		nr	0.96§
Kuethe, 2011 [19]		NL 3	Mean PD20 fall in FEV1.	24	0.75(−0.33 to +1.82)*		0.10(−0.95 to +1.16)†		nr	0.55§
Kuethe, 2011 [19]		NL 3	Lung function, % FEV1 of predicted value.	12	3.6 (−0.2 to 7.5)*		0.5 (−3.3 to 4.3)†		nr	0.29§
Kuethe, 2011 [19]		NL 3	Lung function, % FEV1 of predicted value.	24	2.5 (−1.1 to 6.2)*		0.9 (−2.7 to 4.5)†		nr	0.57§
Kuethe, 2011 [19]		NL 3	Mean p.p.b FENO (3 breath manoeuvres).	12	−2.5(−6.6 to 3.4)*		1.6(−2.8 to 7.8)†		nr	0.44§
Kuethe, 2011 [19]		NL 3	Mean p.p.b FENO (3 breath manoeuvers).	24	−5.1(−9.4 to 0.9)*		−2.9(−8.2 to 3.5)†		nr	0.36§
**Kidney function**										
Denver, 2003 [26]		UK 2	>30 mg/day of urinary albumin excretion = complications.	6	39.2 (16.0 to 200.0)‡		30.5 (14.5 to 147.2)‡		nr	nr
Denver, 2003 [26]		UK 2	Mean urine sodium excretion, mmol/day.	6	178.7 (103.1)	59	177.3 (87.7)	56	1.4 (−33.52 to 36.32)	0.940
Denver, 2003 [26]		UK 2	Mean serum creatinine, µmol/day.	6	117.6 (40.2)	59	114.7 (37.2)	56	2.9 (−11.25 to 17.05)	0.690
**Incontinence**										
Du Moulin, 2007 [24]		NL 1	Frequency of incontinence episodes.	6	5.3 (7.4)	35	9.6 (7.7)	10	−4.3 (−9.67 to 1.07)	0.120
Du Moulin, 2007 [24]		NL 1	Frequency of incontinence episodes.	12	4.8 (7.5)	35	8.6 (5.6)	10	−3.8 (−8.07 to 0.47)	0.080
Du Moulin, 2007 [24]		NL 1	Volume of incontinence episodes, number of pads.	6	5 (3.8)	35	6.4 (3.9)	10	−1.4 (−4.13 to 1.33)	0.310
Du Moulin, 2007 [24]		NL 1	Volume of incontinence episodes, number of pads.	12	4.2 (4.2)	35	5.8 (4.1)	10	−1.6 (−4.5 to 1.3)	0.280
**HIV/AIDS**										
Fairall, 2012 [18]		ZA 1	CD4 count for ART initiation.	12–18	161 (175)	2345	141 (161)	1544	20 (9.29 to 30.71)	0.000
Fairall, 2012 [18]		ZA 2	CD4 count for ART continuation and regimens.	12–18	438 (219)	1733	418 (201)	1691	20 (5.93 to 34.07)	0.005

**Legend.**

Studies are listed in order of increasing length of follow-up, within each category of outcomes.

Abbreviations: US = United States; NL = The Netherlands; UK = United Kingdom; ZA = South Africa; FUP = follow-up; m = months; N = total number of patients per group; SD = standard deviation; WMD = weighted mean difference; CI = confidence intervals; nr = not reported; ART = Antiretroviral Therapy; FENO = Fraction of Exhaled Nitric Oxide; PD20 = provocative dose of methacholine causing a 20% fall in forced expiratory volume in one second (FEV1); LDL = Low Density Lipoprotein; TC = Total Cholesterol; HDL = High Density Lipoprotein; mmol/l = millimoles per litre; µmol/day = micromoles per day; CD4 = t-cell surface glycoprotein CD4; l/min = litre per minute.

^*^ reported mean (90% CIs) for nurse versus general physician: PD20-FEV1, % predictive value-FEV1, p.p.b FENO.

† reported mean (90% CIs) for nurse versus paediatrician: PD20-FEV1, % predictive value-FEV1, p.p.b FENO.

‡ reported the median (interquartile ranges) for nurse and physician groups respectively.

§ reported between nurse/general physician versus nurse/paediatrician.

### Blood Pressure

Five trials provided sufficient quantitative continuous data for meta-analysis (Figure 2). Compared to physician-led care, the pooled WMD revealed a significant SBP-reducing effect of nurse-led care interventions (SBP, mmHg: WMD -4.27, 95%CI -6.31 to -2.23; p<0.0001). The pooled WMD also favoured a DBP-reducing effect of nurse-led care interventions but the confidence intervals crossed the line of no effect (DBP, mmHg: WMD -1.48, 95%CI -3.05 to -0.09; p = 0.06). There was no significant heterogeneity between trials (SBP: I^2^ = 0%, p = 0.53; DBP: I^2^ = 38%, p = 0.19).

### Cholesterol and Triglycerides

Meta-analysis of four trials demonstrated no significant differences between nurse-led care and physician-led care in reducing the mean levels of total cholesterol (TC) at follow up, with no significant heterogeneity between trials (TC, mmol/l: WMD -0.08, 95%CI -0.22 to 0.07, p = 0.29; I^2^ = 0%) (Figure 3). Individual trial estimates showed significantly more patients with nurse-led care had a positive decrease or regression in TC and low density lipoprotein (LDL) levels than did patients in the group of physicians [22]. Other trial estimates showed no significant differences between groups in the reduction of LDL, high density lipoprotein (HDL), TC/HDL ratio or triglycerides [20], [21], [26].

### Glycosylated Haemoglobin Concentration

Meta-analysis of four trials demonstrated no significant differences between nurse-led care and physician-led care in reducing glycosylated haemoglobin concentrations (HbA1c) at follow up, with no significant heterogeneity between trials (HbA1c, %: WMD 0.12, 95%CI -0.13 to 0.37, p = 0.33; I^2^ = 0%) (Figure 3). Similarly, trial estimates showed no significant differences in the number of patients with a positive decrease or regression in blood glucose levels [22].

### Lung and Kidney Function

Individual trial estimates showed no significant differences between groups in various parameters of lung function including measurements of peak flow at six months [28], and PD20, lung function (%FEV1) or FENO either at 12 or 24 months [19] (Table 4). Similarly, there were no significant differences between groups in the parameters of kidney function including the levels of urine sodium excretion and serum creatinine at six months [26]. The reported median (IQR) levels of urinary albumin excretion tested to detect renal complications were higher in the nurse-led care group [UAER, mmol/day: nurse-led care, median 39.2 (IQR 16.0 to 200.0) vs. physician-led care, median 30.5 (IQR 14.5 to 147.2)].

### Cardiac Function

Compared to physician-led care, there were significantly more patients with nurse-led care who had a decrease or regression in the levels of functional exercise capacity, N-terminal pro-brain natriuretic peptide or in the left ventricular end-diastolic volume index [22] (Table 3). There were no significant differences between groups in the levels of C-reactive protein, left atrial size index, and left ventricular mass index or in the ratio of early to late mitral valve flow velocity.

### Incontinence

Individual trial estimates showed no significant differences between groups in the frequency (number and volume) or volume (number of pads) of incontinent episodes at either 6 or 12 months follow-up [24] (Table 4).

### Parkinson’s Disease

Individual trial estimates showed no significant differences between groups in the fractures sustained during study or in the results from the mobility stand-up test at 24 months follow-up [27] (Table 3).

### HIV/AIDS

In one trial, CD4 cell-counts were used as indication for ART initiation (Cohort 1) and ART continuation and management of regimens (Cohort 2) at 12–18 months follow-up [18] (Table 4). Patients receiving nurse-led care had significantly lower CD4 cell-counts compared to patients who received physician-led care.

## Discussion

We systematically evaluated the published evidence for the effects of physician-nurse substitution on clinical parameters in 11 RCTs involving more than 30,000 patients with various conditions. The first important and surprising finding of our review is that the number of studies in this area is increasing slowly and studies continue to be of poor quality despite evidence reports published ten years ago [7], [8]. There is also a surprising low volume of literature reporting the outcomes of interest for this review. Most of the studies tend to report more process of care than clinical parameters. There were only three outcomes for which we could quantify the intervention effects using meta-analyses and these comprised a maximum of five studies each. The studies were also generally small. Only 3 of the 11 RCTs had more than 200 patients per arm. Of the studies pooled in meta-analyses, only one had more than 200 patients in each group. Furthermore, no study fulfilled the assessed set of methodological quality criteria. Nearly half of the 11 RCTs suffered from attrition of significant concern (≥20% attrition) and selection (i.e. lack of, or unclear, allocation concealment) biases and only a few were sufficiently powered to detect a true effect. Although we could not investigate the possibility of publication bias, we cannot rule it out since our review was limited to the published literature. Lastly, more than half of the evidence reviewed has been conducted in Europe, mainly the Netherlands. Our review shows the best available evidence however.

The evidence represents interventions for which nurses trained to provide care in various settings and for a wide range of complex conditions. In most cases, (82%), this required specialised skills and the use of guidelines. This suggests that the level of skills may be critical for the success of disease management when physician-nurse substitution takes place. The level of substitution did not seem consistent across studies however. Trials employed nurses of various qualifications and the tasks performed varied regardless of the level of training. Moreover, despite possessing some level of advanced skills, nurses required support or communication with the physicians for various tasks. Thus it was difficult to explore and identify patterns of potential influences of this criterion on the outcomes. On one hand, all studies included in the meta-analyses employed nurse practitioners (with or without further degrees), and the direction of effect remained after systematic exclusion of each trial. On the other hand, studies for which data could not be pooled involved licensed nurses, registered nurses, or nurse practitioners (with or without further degrees). Perhaps the development of nurse-led clinics may be a more appealing strategy to allow nurses to establish full clinical autonomy. The reporting of other clinicians’ characteristics (e.g. nurse-physician-patient ratios, and years of experience) also remains insufficient despite previous findings [7], [8].

There are gaps in the current evidence which merit consideration in further studies. Nurses’ roles and the level of experience required to qualify for substitution need a better definition of boundaries and task allocation in clinical practice. In addition, future research should generate more methodologically sound studies. Consistent and complete reporting of the aforementioned characteristics could improve the understanding and identification of the optimal benefits of this strategy. In spite of these limitations and heterogeneity, our meta-analyses demonstrate a statistically significant systolic blood pressure-reducing effect of nurse-led care (delivered by nurse practitioners) compared to physician-led care but no significant differences in reducing diastolic blood pressure, total cholesterol and glycosylated haemoglobin. Results from the other 32 individual trial estimates reported in nine of the trials suggest that nurse-led care (by nurses with various titles) may be similarly (26 estimates) or more (6 estimates) effective than physicians at managing the variety of clinical parameters evaluated in our review.

### Strengths and Limitations of the Review

To our knowledge this is the first systematic review with a focus on clinical parameters in patients undergoing care with the implementation of physician-nurse substitution in primary care. It benefits from a thorough assessment and critical appraisal of RCTs which are at lower risk of bias [29], [30] than observational studies and allow the identification of causal relationships. It also presents the corresponding individual trial estimates and 95% confidence intervals for outcome data for which meta-analyses were not possible. A limitation of our review however, is the small number of studies that met the inclusion criteria, hence threatening the robustness of findings. This may be explained by the fact that the number of studies in this area is increasing slowly, we did not search for grey literature and were unable to identify many studies that reported the outcomes of interest. In addition, almost every study investigated only one condition, resulting in divergent reporting of outcomes that were unique to each study. It was difficult thus to study every area in depth and biases may have arisen from the over-representation of nurse-led care in specialised areas. Although many different clinical parameters were reported among the 11 RCTs, the small number of studies with sufficient and appropriate data limited meta-analyses to three outcomes. This also limited the exploration of effects in pre-specified subgroup analyses. The most probable small study bias thus accounted for the observed effects is publication bias (i.e. when the results of small negative studies are less likely to be published than small studies with positive results). A further potential limitation is the inclusion of publications in English only. We did however use comprehensive searches and screened the reference lists of included studies and relevant reviews (some in foreign languages). We did not contact authors to further obtain or clarify missing information.

## Conclusion

Trained nurses appeared to be better than physicians at lowering systolic blood pressure but similar at lowering diastolic blood pressure, total cholesterol or glycosylated haemoglobin. While only a few individual trial estimates of 32 clinical parameters (e.g. kidney and lung function) favoured nurse-led care, there is insufficient evidence to conclude that nurse-led care leads to better outcomes of clinical parameters than physician-led care. The main limiting factor is the insufficient quantity of studies using good quality methods and reporting the same outcome. The current evidence also shows disease specific interventions from a small selection of healthcare systems. In order to provide more general conclusions, far more good quality trials in larger numbers of patients need to be carried out. Furthermore, additional studies should map clinicians’ characteristics, including the wider range of nurse care and tasks provided in many countries and the various levels of training and clinical autonomy.

## Supporting Information

Table S1 Search strategy in Ovid Medline*. *Similar search strategies were performed and run in EMBASE, The Cochrane Library of Systematic Reviews and CINAHL. All included specific search filters for RCTs.
(DOCX)

Table S2 Studies excluded from the review based on appraisal of full-text articles.
(DOCX)

Table S3 Participants, interventions and outcomes, in the included studies. Studies are listed by year (y) of publication, in decreasing order. Abbreviations: US = United States; NL = The Netherlands; UK = United Kingdom; ZA = South Africa; RU = Russia; RCT = Randomised Controlled Trial; cRCT = Cluster Randomised Controlled Trial; NR = Not Reported; ART = Antiretroviral Therapy; HbA1c = Haemoglobin; BP = Blood Pressure; TC = Total Cholesterol; GH = Glycosylated Haemoglobin; CD4 = t-cell surface glycoprotein CD4; HDL = High Density Lipoprotein levels; LDL = Low Density Lipoprotein; PD20 = provocative dose of methacholine causing a 20% fall in forced expiratory volume in one second (FEV1); FENO = Fraction of Exhaled Nitric Oxide. * start and end year when studies were conducted.
(DOCX)

Checklist S1 **PRISMA Checklist.**
(DOC)
