# Supplementary material for: Effects of Physician-Nurse Substitution on Clinical Parameters: A Systematic Review and Meta-Analysis
Source: PLoS One. 2014 Feb 24;9(2):e89181. doi: 10.1371/journal.pone.0089181 (PMC3933531; doi:10.1371/journal.pone.0089181)
Supplement: Table S2 — Studies excluded from the review based on appraisal of full-text articles. (DOCX) [file pone.0089181.s002.docx]

**Table S2. Studies excluded from the review based on appraisal of full-text articles.**

| **Item, #** | **Reference to study** | **Reason for exclusion** |
| --- | --- | --- |
| [1] | Blanchard MR, Waterreus A, Mann AH. Can a brief intervention have a longer-term benefit? The case of the research nurse and depressed older people in the community. Int J Geriatr Psychiatry. 1999; 14:733-8. | Cohort study and multidisciplinary team approach. |
| [2] | Blanchard MR, Waterreus A, Mann AH. The effect of primary care nurse intervention upon older people screened as depressed. Int J Geriatr Psychiatry. 1995; 10:289-98. | Nurse working in close collaboration with other clinicians. |
| [3] | Campbell NC, Ritchie LD, Thain J, Deans HG, Rawles JM, Squair JL. Secondary prevention in coronary heart disease: a randomised trial of nurse led clinics in primary care. Heart. 1998c; 80:447-52. | Outcomes of interest not reported. |
| [4] | Campbell NC, Thain J, Deans HG, Ritchie LD, Rawles JM, Squair JL. Secondary prevention clinics for coronary heart disease: randomised trial of effect on health. BMJ. 1998b; 316:1434-7. | Outcomes of interest not reported. |
| [5] | Campbell NC, Thain J, Deans HG, Ritchie LD, Rawles JM. Secondary prevention in coronary heart disease: baseline survey of provision in general practice. BMJ. 1998a; 316:1430-4. | Outcomes of interest not reported. |
| [6] | Cave AJ, Wright A, Dorrett J, McErlain M. Evaluation of a nurse-run asthma clinic in general practice. Primary Care Respiratory Journal. 2001; 10:65-8. | Not an intervention comparison between nurses and physicians. |
| [7] | Chan D, Harris S, Roderick P, Brown D, Patel P. A randomised controlled trial of structured nurse-led outpatient clinic follow-up for dyspeptic patients after direct access gastroscopy. BMC Gastroenterol. 2009; 9:12. | Outcomes of interest not reported. |
| [8] | Delaney EK, Murchie P, Lee AJ, Ritchie LD, Campbell NC. Secondary prevention clinics for coronary heart disease: a 10-year follow-up of a randomised controlled trial in primary care. Heart. 2008; 94:1419-23. | Outcomes of interest not reported. |
| [9] | Dierick-Van Daele ATM, Metsemakers JFM, Derckx EWCC, Spreeuwenberg C, Vrijhoef HJM. Nurse practitioners substituting for general practitioners: Randomized controlled trial. Journal of Advanced Nursing. 2009; 65:391-401. | Outcomes of interest not reported. |
| [10] | Dierick-Van Daele ATM, Steuten LMG, Metsemakers JFM, Derckx EWCC, Spreeuwenberg C, Vrijhoef HJM. Economic evaluation of nurse practitioners versus GPs in treating common conditions. British Journal of General Practice. 2010; 60:28-33. | Outcomes of interest not reported. |
| [11] | Flynn BC. The effectiveness of nurse clinicians' service delivery. Am J Public Health. 1974; 64:604-11. | Outcomes of interest not reported. |
| [12] | Hemani A, Rastegar DA, Hill C, al-Ibrahim MS. A comparison of resource utilization in nurse practitioners and physicians. Eff Clin Pract. 1999; 2:258-65. | Outcomes of interest not reported. |
| [13] | Hesselink AE, Penninx BW, van der Windt DA, van Duin BJ, de Vries P, Twisk JW, et al. Effectiveness of an education programme by a general practice assistant for asthma and COPD patients: results from a randomised controlled trial. Patient Educ Couns. 2004; 55:121-8. | Outcomes of interest not reported. |
| [14] | Hollinghurst S, Horrocks S, Anderson E, Salisbury C. Comparing the cost of nurse practitioners and GPs in primary care: modelling economic data from randomised trials. British Journal of General Practice. 2006; 56:530-5. | Outcomes of interest not reported. |
| [15] | Kernick D, Cox A, Powell R, Reinhold D, Sawkins J, Warin A. A cost consequence study of the impact of a dermatology-trained practice nurse on the quality of life of primary care patients with eczema and psoriasis. British Journal of General Practice. 2000; 50:555-8. | Outcomes of interest not reported. |
| [16] | Kernick D, Powell R, Reinhold D. A pragmatic randomised controlled trial of an asthma nurse in general practice. Primary Care Respiratory Journal. 2002; 11:6-8. | Outcomes of interest not reported. |
| [17] | Kinnersley P, Anderson E, Parry K, Clement J, Archard L, Turton P, et al. Randomised controlled trial of nurse practitioner versus general practitioner care for patients requesting "same day" consultations in primary care. BMJ. 2000; 320:1043-8. | Outcomes of interest not reported. |
| [18] | Krein SL, Klamerus ML, Vijan S, Lee JL, Fitzgerald JT, Pawlow A, et al. Case management for patients with poorly controlled diabetes: a randomized trial. Am J Med. 2004; 116:732-9. | Nurse working in close collaboration with other clinicians based on a chronic care model. |
| [19] | Lapointe F, Lepage S, Larrivee L, Maheux P. Surveillance and treatment of dyslipidemia in the post-infarct patient: can a nurse-led management approach make a difference? Can J Cardiol. 2006; 22:761-7. | Intervention (telephone) not of interest for this review and not part of usual care interventions of physicians. |
| [20] | Leenders F, Beusmans G, Swerts H, editors. A practice nurse for patients with cardiovascular disease, an explorative study2006. | Report in Dutch. Version of article in English was not found. |
| [21] | Lewis CE, Resnik BA, Schmidt G, Waxman D. Activities, events and outcomes in ambulatory patient care. N Engl J Med. 1969; 280:645-9. | Not at RCT. |
| [22] | Lewis CE, Resnik BA. Nurse clinics and progressive ambulatory patient care. N Engl J Med. 1967; 277:1236-41. | Outcomes of interest not reported |
| [23] | Murchie P, Campbell NC, Ritchie LD, Deans HG, Thain J. Effects of secondary prevention clinics on health status in patients with coronary heart disease: 4 year follow-up of a randomized trial in primary care. Fam Pract. 2004; 21:567-74. | Outcomes of interest not reported. |
| [24] | Murchie P, Campbell NC, Ritchie LD, Simpson JA, Thain J. Secondary prevention clinics for coronary heart disease: four year follow up of a randomised controlled trial in primary care. BMJ. 2003; 326:84. | Outcomes of interest not reported. |
| [25] | Raftery JP, Yao GL, Murchie P, Campbell NC, Ritchie LD. Cost effectiveness of nurse led secondary prevention clinics for coronary heart disease in primary care: follow up of a randomised controlled trial. BMJ. 2005; 330:707. | Outcomes of interest not reported. |
| [26] | Sackett DL, Spitzer WO, Gent M, Roberts RS. The Burlington randomized trial of the nurse practitioner: health outcomes of patients. Ann Intern Med. 1974; 80:137-42. | No real substitution. At least 30% of patients in both groups were seen by the physicians at the end of study and data not split into mutually exclusive groups. |
| [27] | Shum C, Humphreys A, Wheeler D, Cochrane MA, Skoda S, Clement S. Nurse management of patients with minor illnesses in general practice: multicentre, randomised controlled trial. BMJ. 2000; 320:1038-43. | Outcomes of interest not reported. |
| [28] | Spitzer WO, Sackett DL, Sibley JC, Roberts RS, Gent M, Kergin DJ, et al. The Burlington randomized trial of the nurse practitioner. N Engl J Med. 1974; 290:251-6. | No real substitution. At least 30% of patients in both groups were seen by the physicians at the end of study and data not split into mutually exclusive groups. |
| [29] | Tonstad S, Alm CS, Sandvik E. Effect of nurse counselling on metabolic risk factors in patients with mild hypertension: a randomised controlled trial. Eur J Cardiovasc Nurs. 2007; 6:160-4. | No real substitution. In both experimental and control groups the nurse provides interventions at different stages of care. |
| [30] | Van Son L, Vrijhoef, H. Supporting the general practitioner. A randomized controlled trial investigation the effects of a practice nurse on asthma, COPD, and diabetes. 2004; Huisarts en wetenschap:15-21. | Report in Dutch. Version of article in English was not found. |
| [31] | Venning P, Durie A, Roland M, Roberts C, Leese B. Randomised controlled trial comparing cost effectiveness of general practitioners and nurse practitioners in primary care. BMJ. 2000; 320:1048-53. | Outcomes of interest not reported. |
| [32] | Williams KS, Assassa RP, Cooper NJ, Turner DA, Shaw C, Abrams KR, et al. Clinical and cost-effectiveness of a new nurse-led continence service: a randomised controlled trial. Br J Gen Pract. 2005; 55:696-703. | Nurse working in close collaboration with other clinicians. Control group received care from nurses, physicians and specialists and data not split into mutually exclusive groups. |
| [33] | Winter C. Quality health care: patient assessment [MSc]. Long Beach, CA: California State University; 1981. | Outcomes of interest not reported. |
