# Supplementary material for: Effects of Physician-Nurse Substitution on Clinical Parameters: A Systematic Review and Meta-Analysis
Source: PLoS One. 2014 Feb 24;9(2):e89181. doi: 10.1371/journal.pone.0089181 (PMC3933531; doi:10.1371/journal.pone.0089181)
Supplement: Table S3 — Participants, interventions and outcomes, in the included studies. Studies are listed by year (y) of publication, in decreasing order. Abbreviations: US = United States; NL = The Netherlands; UK = United Kingdom; ZA = South Africa; RU = Russia; RCT = Randomised Controlled Trial; cRCT = Cluster Randomised Controlled Trial; NR = Not Reported; ART = Antiretroviral Therapy; HbA1c = Haemoglobin; BP = Blood Pressure; TC = Total Cholesterol; GH = Glycosylated Haemoglobin; CD4 = t-cell surface glycoprotein CD4; HDL = High Density Lipoprotein levels; LDL = Low Density Lipoprotein; PD20 = provocative dose of methacholine causing a 20% fall in forced expiratory volume in one second (FEV1); FENO = Fraction of Exhaled Nitric Oxide. * start and end year when studies were conducted. (DOCX) [file pone.0089181.s003.docx]

**Table S3. Participants, interventions and outcomes, in the included studies.**

| **Study** | | | **Participants** | | **Intervention** | | | **Outcomes** |
| --- | --- | --- | --- | --- | --- | --- | --- | --- |
| **First author, year** | **Location** | **Design, period**^*^ | **Included** | **Excluded** | **Care delivered** | **Clinical autonomy** | **Follow-up, months** | **Reported** |
| Fairall, 2012 [18]. | ZA 2 | cRCT (cohort 2), 2008-2010 | Adults with HIV who received ART for at least six months with ongoing treatment at the time of enrolment; clinics with more than 100 eligible patients used a random sample taken electronically (proportional to the total number of eligible patients); in other clinics all eligible patients were included. | Patients who did not return to their clinic after enrolment or who were potentially exposed to the intervention; deaths before trial started or were relocated to another clinic. | Nurse-led care based on African guidelines. Middle nurse managers trained to assume responsibility for ART: i.e. assess and prepare patients, initiate, monitor and prescribe ART or referral to physicians for ART initiation and re-prescriptions. Physicians referred patients diagnosed with HIV to nurse-led clinic to establish eligibility for ART. | For phase 2 and phase 3: same as in cohort 1. | 18 | CD4 counts for ART continuation and regimens. |
| Fairall, 2012 [18]. | ZA 1 | cRCT (cohort 1), 2008-2010 | Adults with HIV participating in an ART program who had CD4 counts of ≤350/µl and had not yet started ART: either eligible for ART (if CD4 counts were ≤200 cells per μL) or likely to become eligible during the trial (if CD4 counts were 201-350 cells per μL). | Patients who did not return to their clinic after enrolment because they needed to visit a clinic more than once to initiate ART after obtaining CD4 results; who started ART before trial started, who deaths before trial started or were relocated to another clinic. | Nurse-led care based on African guidelines. Middle nurse managers trained to assume responsibility for ART: i.e. assess and prepare patients; initiation, monitoring and prescribing ART or referral to physicians for ART initiation and re-prescriptions. Physicians referred patients diagnosed with HIV to nurse-led clinic to establish eligibility for ART. | For phase 2: nurses assumed responsibility for repeating ART prescriptions in stable (median, m: 3.5, range: 1-35) patients. For phase 3: nurses assumed responsibility for initiating ART in selected patients (median, m: 30.5, range: 0-32) and nurses referred patients who did not meet above criteria. | 16-18 | CD4 counts for ART initiation. |
| Houweling, 2011 [20]. | NL 4 | RCT, period NR. | Patients under treatment and under medication for diabetes mellitus type 2, with HbA1c measurements within the last three years. | Patients with diabetes mellitus type 2 not being treated in primary care setting, inability to participate because of age, comorbidities or -in the opinion of the general practitioner- whoever was not willing to return for follow-up. | Practice nurse with one week training in diabetes mellitus to manage transferred patients based on guidelines. | Full responsibility. | 14 | BP, TC, GH, TC/HDL ratio. |
| Kuethe, 2011 [19]. | NL 3 | RCT, 2006-2008. | Patients 6-16 years old with moderate and stable asthma, in treatment of inhalative corticosteroids at least nine months before the start of the study, informed consent. | Patients not able to perform lung function tests, or who had other chronic diseases. | Asthma nurse to manage patients based on guidelines. | Nurses needed support from/or short communication with paediatrician. | 24 | Lung function: PD20, %FEV1, FENO. |
| Voogdt-Pruis 2010 [21]. | NL 2 | RCT, 2006-2007. | Patients 30-74 years of age, with cardiovascular disease or hypertension and/or hypercholesterolemia, with at least 10% in 10-year risk of cardiovascular disease; risk due to systolic blood pressure of ≥140 or total cholesterol of ≥6.5 mmol/l within the previous six months. | Patients visiting specialist in cardiovascular disease more than once per year, diabetes mellitus, severe comorbidities. | Nurse-led care based on Dutch guidelines for cardiovascular risk management. Advance practice nurse managed cardiovascular risk including primary and secondary prevention. | NR. | 12 | BP, TC. |
| Andryukhin 2010 [22] | RU1 | RCT, 2006-2009. | Patients of at least 50 years of age with Heart Failure with preserved ejection fraction, informed consent. | Patients with blood pressure of <90/60 mmHg or >160/100 mmHg, under optimal antihypertensive therapy, acute coronary syndrome within previous six months, significant valvular stenosis, insulin diabetes mellitus dependent, confirmed chronic obstructive pulmonary disease, conditions limiting participation in the rehabilitation (see reference for more details). | Nurse-led care based on Russian National guidelines. Nurses with special degree in patient education obtained in joint course: patient education, treatment and exercise training information and counselling. | Prescription of medication and non-pharmacological measures (diet, alcohol intake, weight reduction, smoking cessation, activity and exercise training) provided by physician. | 6, 18 | TC, GH, LDL, cardiac function and inflammation. |
| Hiss, 2007 [23]. | US 2 | RCT, period NR. | Patients of at least 18 years of age with diabetes mellitus type 2. | NR. | Diabetes nurse to manage patients based on guidelines (evaluation/examination; identification/discussion of short/long term goals; action plan, proactive/continuous follow-up; communicating results to patient and physician; blood samples for urine analysis). | Nurses received support from physicians as the physicians of record to discuss patients’ records; nurses also developed an action plan together with the physicians. | 6 | BP, TC, GH. |
| Du Moulin, 2007 [24]. | NL 1 | cRCT, period NR. | Female community-dwelling patients, older than 18 years with any form of incontinence, presenting with urinary incontinence for the first time, informed consent. | Patients with bacteriuria, with post-void residual urine volume of more than100 ml, who gave birth within three months before study, with bladder cancer, renal disease, uterus prolapse or past introitus. | Nurse-led care based on protocols. Registered nurse specialist in incontinence: management and patient education, to provide advice on lifestyle, treatment and use of pads; and referred patients to urologist. | Reporting of findings to physician after each visit; urinalysis completed by physician. | 12 | Incontinence: frequency and volume. |
| Denver, 2003 [26]. | UK 2 | RCT, 2000-2001. | Patients with diabetes mellitus type 2, previous diagnosis of hypertension, or who were in receipt of blood pressure lowering treatment. | Patients with life-threatening comorbidities requiring intensive management. | Hypertension nurse care based on clinical guidelines. | No (details NR). | 6 | BP, TC, HG, HDL, triglycerides, kidney function. |
| Jarman, 2002 [27]. | UK 1 | RCT, 1996-1999. | Patients with Parkinson’s Disease taking one or more anti-Parkinson drugs, informed consent. | Patients younger than17 years of age, severe mental illness, sufficient cognitive impairment. | Community nurse with a course in Parkinson’s Disease: advised physicians, provided patient counselling and education, treatment information and monitoring; reporting to physicians, instigating respite, day hospital care and discharge; assessment of patient social security, liaison with multidisciplinary primary care teams for ongoing assessment and therapy. | Nurses were under guidance of a nurse manager but had advisory position to physicians with whom patients’ records were discussed. | 24 | Stand-up and mobility (tests). |
| Mundinger, 2000 [25, 28]. | US 1 | RCT, 1995-1997. | Patients with previous diagnosis of asthma, diabetes mellitus, hypertension, or requesting urgent visits. | Patients having a current primary care provider, planned to leave the area within the following six months, or failed to keep their initial appointment. | Community nurse practitioners provided ambulatory primary care, 24-hr call, made independent decisions for referral to specialists and hospitalisations. | Independent decisions for referrals to specialists and hospitalisations but unclear about the rest e.g. prescriptions. | 6-12 (phase I), 24 (phase II) | BP, GH, peak flow. |

**Legend.**

Studies are listed by year (y) of publication, in decreasing order.

Abbreviations: US = United States; NL = The Netherlands; UK = United Kingdom; ZA = South Africa; RU = Russia; RCT = Randomised Controlled Trial; cRCT = Cluster Randomised Controlled Trial; NR = Not Reported; ART = Antiretroviral Therapy; HbA1c = Haemoglobin; BP = Blood Pressure; TC = Total Cholesterol; GH = Glycosylated Haemoglobin; CD4 = t-cell surface glycoprotein CD4; HDL = High Density Lipoprotein levels; LDL = Low Density Lipoprotein; PD20 = provocative dose of methacholine causing a 20% fall in forced expiratory volume in one second (FEV1); FENO = Fraction of Exhaled Nitric Oxide.

^*^ start and end year when studies were conducted.
